# *Bruguiera gymnorhiza* (L.) Lam. at the Forefront of Pharma to Confront Zika Virus and Microbial Infections—An In Vitro and In Silico Perspective

**DOI:** 10.3390/molecules26195768

**Published:** 2021-09-23

**Authors:** Nabeelah Bibi Sadeer, Juliano G. Haddad, Mohammed Oday Ezzat, Philippe Desprès, Hassan H. Abdallah, Gokhan Zengin, Ahmet Uysal, Chaker El Kalamouni, Monica Gallo, Domenico Montesano, Mohamad Fawzi Mahomoodally

**Affiliations:** 1 Department of Health Sciences, Faculty of Medicine and Health Sciences, University of Mauritius, Réduit 80837, Mauritius; nabeelah.sadeer1@umail.uom.ac.mu; 2 Unité Mixte Processus Infectieux en Milieu Insulaire Tropical, Plateforme Technologique CYROI, Université de la Réunion, INSERM U1187, CNRS UMR 9192, IRD UMR 249, 94791 Sainte Clotilde, La Réunion, France; juliano.haddad@univ-reunion.fr (J.G.H.); philippe.despres@univ-reunion.fr (P.D.); chaker.el-kalamouni@univ-reunion.fr (C.E.K.); 3 Department of Chemistry, College of Education for Women, University of Anbar, Ramadi 31001, Iraq; edw.mohamed_oday@uoanbar.edu.iq; 4 Chemistry Department, College of Education, Salahaddin University-Erbil, Erbil 44001, Iraq; hassan.abdullah@su.edu.krd; 5 Department of Biology, Science Faculty, Selcuk University, Campus, 42130 Konya, Turkey; gokhanzengin@selcuk.edu.tr; 6 Department of Medicinal Laboratory, Vocational School of Health Services, Selcuk University, 42130 Konya, Turkey; ahuysal@selcuk.edu.tr; 7 Department of Molecular Medicine and Medical Biotechnology, University of Naples Federico II, via Pansini 5, 80131 Naples, Italy; mongallo@unina.it; 8 Department of Pharmacy, University of Naples Federico II, via D. Montesano 49, 80131 Naples, Italy

**Keywords:** mangrove plants, antiviral, antimicrobial, envelope protein, ADME, pharmacokinetics

## Abstract

The recent emergence of Zika virus (ZIKV) in Brazil and the increasing resistance developed by pathogenic bacteria to nearly all existing antibiotics should be taken as a wakeup call for the international authority as this represents a risk for global public health. The lack of antiviral drugs and effective antibiotics on the market triggers the need to search for safe therapeutics from medicinal plants to fight viral and microbial infections. In the present study, we investigated whether a mangrove plant, *Bruguiera gymnorhiza* (L.) Lam. (*B. gymnorhiza*) collected in Mauritius, possesses antimicrobial and antibiotic potentiating abilities and exerts anti-ZIKV activity at non-cytotoxic doses. Microorganisms *Escherichia coli* ATCC 25922, *Pseudomonas aeruginosa* ATCC 27853, *Klebsiella pneumoniae* ATCC 70603, methicillin-resistant *Staphylococcus aureus* ATCC 43300 (MRSA), *Salmonella enteritidis* ATCC 13076, *Sarcina lutea* ATCC 9341, *Proteus mirabilis* ATCC 25933, *Bacillus cereus* ATCC 11778 and *Candida albicans* ATCC 26555 were used to evaluate the antimicrobial properties. Ciprofloxacin, chloramphenicol and streptomycin antibiotics were used for assessing antibiotic potentiating activity. ZIKV^MC-MR766NIID^ (ZIKV^GFP^) was used for assessing anti-ZIKV activity. In silico docking (Autodock 4) and ADME (SwissADME) analyses were performed on collected data. Antimicrobial results revealed that *Bruguiera* twig ethyl acetate (BTE) was the most potent extract inhibiting the growth of all nine microbes tested, with minimum inhibitory concentrations ranging from 0.19–0.39 mg/mL. BTE showed partial synergy effects against MRSA and *Pseudomonas aeruginosa* when applied in combination with streptomycin and ciprofloxacin, respectively. By using a recombinant ZIKV-expressing reporter GFP protein, we identified both *Bruguiera* root aqueous and *Bruguiera* fruit aqueous extracts as potent inhibitors of ZIKV infection in human epithelial A549 cells. The mechanisms by which such extracts prevented ZIKV infection are linked to the inability of the virus to bind to the host cell surface. In silico docking showed that ZIKV E protein, which is involved in cell receptor binding, could be a target for cryptochlorogenic acid, a chemical compound identified in *B. gymnorhiza*. From ADME results, cryptochlorogenic acid is predicted to be not orally bioavailable because it is too polar. Scientific data collected in this present work can open a new avenue for the development of potential inhibitors from *B. gymnorhiza* to fight ZIKV and microbial infections in the future.

## 1. Introduction

The continuous rise in numerous resistant bacterial and viral infections, while eluding successful clinical care, has become a major global threat. Unless tackled, infectious diseases promise a future with increasing healthcare expenditures, low life expectancy and a debilitated world population. The rise in drug-resistant microbial and viral infections has triggered the need to search for novel alternative and safer therapeutic agents [1,2]. Bacteria are rapidly developing resistance to existing antibiotics due to over-prescription. Introducing new antibiotics into the market to overcome the ongoing threat of antimicrobial resistance would seem in vain since bacteria have an infinite capacity to adapt to and resist new drugs [3]. Consequently, to consider the problem from a new angle, the effectiveness of existing antibiotics can be enhanced by potentiating them with prospective natural compounds. Accumulating evidence has acknowledged that plants harbor a diverse array of secondary metabolites, namely tannins, alkaloids, phenolic acids, terpenoids, flavonoids and saponins, among others, exhibiting promising in vitro antimicrobial activities [4]. Ellagic acid and tannic acid are known to be effective potentiators of a number of antibiotics including novobiocin, chlorobiocin, coumermycin, fisidic acid and rifampicin [5]. Numerous plant species, not limited to *Piper betle* L., *Psiadia arguta* Pers., *Pimenta dioica* L. [6], *Cordia verbenacea* DC. [7] and *Plectranthus amboinicus* L. [8], exerted significant synergistic effects when combined with gentamicin.

While COVID-19 is currently dominating the headlines, an under-reported virus that once was spread to a swathe of countries in the past has recently re-emerged—the Zika virus (ZIKV). The Ministry of Health in Brazil has reported 579 new ZIKV cases between December 2019 and February 2020 [9,10]. First identified in Uganda in 1947 from rhesus monkeys, the ZIKV, particularly vectored by the yellow fever mosquito, *Aedes aegypti*, is a flavivirus originating from the *Flaviviridae* family [11]. The virus can be transmitted sexually, via blood transfusions, via organ transportation and from pregnant women to their fetuses [12,13]. It is argued that the recent ZIKV re-emergence is due to a lack of interest from researchers since the virus affects mostly poor nations of the world and simultaneously the situation is not considered as urgent enough for pharmaceutical industries to make profits [13]. However, with these new reported cases in Brazil, it is high time that emerging infectious diseases including ZIKV be rescued from their dangerous oblivion in order to prevent future outbreaks.

Recently, a series of papers from different groups have tried to revive this flagging research topic. Medicinal plants have been used as a remedy against numerous ailments including infectious diseases for centuries and still represent an exceptional source of potential antiviral compounds against flaviviruses [14]. Studies demonstrated that plant-derived natural products such as abietane diterpenoids [15], gossypol, curcumin, digitonin and conessine exhibited strong inhibitory activity against ZIKV strains [16]. Crenatoside identified from *Tecoma stans* var. *stans* displayed better anti-ZIKV activity (EC50: 34.78 µM) than the positive control ribavirin (EC50: 386.84 µM) [11]. Lipophilic ethers from citrus fruits present the ability to selectively inhibit ZIKV replication in human breast carcinoma (4T1) cells [17]. The study of Acquadro et al. reported that ellagic acid identified from *Punica granatum* L. can be used as a future therapeutic agent against ZIKV [18]. From the aforementioned studies, since no anti-ZIKV drugs and effective vaccines have been developed yet, and treatments are only palliative, searching for anti-ZIKV candidate compounds from medicinal plants should therefore be considered as a pressing need with the aim to prevent and treat this emerging infection.

Considering the great concern in tackling these pathological urgencies and with the fact that medicinal plants can come to the rescue, the main aim of this present study is therefore to screen an important halophyte, *Bruguiera gymnorhiza* (L.) Lam. (*B. gymnorhiza*), growing along the coastlines of tropical countries including Mauritius for its antimicrobial, antibiotic potentiating and antiviral activities. Several lines of evidence have shown that *B. gymnorhiza* is used as a traditional medicine against diarrhea, viral fever, malaria, eye disease, hypertension, diabetes, stings of toxic lagoon fishes, intestinal worms and gastrointestinal disorders [19]. There is, as yet, no scientific report on the screening of *B. gymnorhiza* on antibiotic potentiating and anti-ZIKV activities. Our second-to-none research work is thus to address such a research gap and contribute to this rising health crisis that has emerged.

## 2. Results and Discussion

### 2.1. Preparation of Plant Extracts for Assays

For sample identification, the following codes were used: BLM: *Bruguiera* leaf methanolic; BRM: *Bruguiera* root methanolic; BTM: *Bruguiera* twig methanolic; BFM: *Bruguiera* fruit methanolic; BLD: *Bruguiera* leaf decoction; BRD: *Bruguiera* root decoction; BTD: *Bruguiera* twig decoction; BFD: *Bruguiera* fruit decoction; BLA: *Bruguiera* leaf aqueous; BRA: *Bruguiera* root aqueous; BTA: *Bruguiera* twig aqueous; BFA: *Bruguiera* fruit aqueous: BLE: *Bruguiera* leaf ethyl acetate; BRE: *Bruguiera* root ethyl acetate; BTE: *Bruguiera* twig ethyl acetate; and BFE: *Bruguiera* fruit ethyl acetate.

The respective yields obtained during the preparation of extracts were calculated and are recorded in Table 1. The highest yield of *B. gymnorhiza* was obtained with BLD (18.70%) followed by BFM (14.28%) and BLM (12.62%). The least effective extraction solvent was ethyl acetate since the yields of ethyl acetate extracts of *B. gymnorhiza* were 0.76–8.06%. High temperatures may have caused the breakage of bonds that exist between analytes and the plant matrix, resulting in a high extraction yield. However, ethyl acetate showed poor extraction efficiency, which could be due to its low polarity.

### 2.2. Antimicrobial Activity

The results of antimicrobial activity are summarized in Table 2. According to the results obtained from decoction extracts, BFD was the most effective extract since it inhibited all the tested strains successfully, with MIC50 values ranging from 0.78–3.12 mg/mL. The growth of *Pseudomonas aeruginosa* was highly affected by this extract (0.78 mg/mL), followed by *Klebsiella pneumoniae*, MRSA, *Salmonella enteritidis, Sarcina lutea* and *Proteus mirabilis* at a dose of 1.56 mg/mL. While MIC was determined as 3.12 mg/mL for the *Candida albicans* strain, the BRD extract was less effective at a dosage of 6.25 mg/mL. BTD inhibited the growth of only three of the tested microorganisms, namely *Pseudomonas aeruginosa*, MRSA and *Proteus mirabilis*, with MIC values of 3.25, 0.78 and 3.12 mg/mL, respectively. BLD proved to be a weak antibacterial inhibitor except for MRSA, revealing a MIC value of 6.25 mg/mL. On the other hand, BRD was significantly effective on MRSA and *Proteus mirabilis* with 0.39 and 0.78 mg/mL MIC values, respectively (Table 2). However, BRD was defined as a weak antifungal candidate with 6.25 mg/mL as the MIC value.

Among the extracts prepared by maceration (water), only BFA displayed a wider antimicrobial activity, showing inhibition with six bacteria. Interestingly, no antimicrobial potential was reported with BLA. On the other hand, aqueous root (BRA) extract revealed significant antibacterial activity against the MRSA strain at a dose of 0.39 mg/mL. It was observed that twig aqueous extract had no effect on all tested microorganisms except for MRSA, which is reported as weak activity. However, BFA was very effective on MRSA at a dose of 0.39 mg/mL and followed by *Proteus mirabilis* with a concentration of 1.56 mg/mL (Table 2). It was also observed that maceration extracts of *B. gymnorhiza* parts showed no antifungal activities.

Among the different types of extracts prepared, ethyl acetate extracts displayed the most interesting antimicrobial activity since their MIC values were lower, ranging from 0.19–3.12 mg/mL, followed by methanolic extracts (MIC: 0.39–3.12 mg/mL), decoction and aqueous extracts (MIC: 0.39–6.25 mg/mL). Among all extracts prepared, BTE was the most effective inhibitor against all tested strains, reporting the lowest MIC values (0.19–0.39 mg/mL). Therefore, this extract can be considered as a broad-spectrum antimicrobial agent managing numerous severe infections. The significant antimicrobial activity displayed by the ethyl acetate extracts could be linked with their phytochemical profiles. In other words, the combination of compounds present in these types of extracts could have created synergistic effects resulting in such an observation. Interestingly, it is important to highlight that all extracts of *B. gymnorhiza*, with the exception of BLA, displayed significant anti-MRSA activities. MRSA is a contagious bacterial infection affecting many patients in contact with the hospital setting [20]. It is associated with several dermatological and life-threatening bloodstream infections, and also pneumonia and surgical site infections [21].

### 2.3. Antibiotic Potentiating Activity

Potentiation or synergism occurs when a combination of two or more drugs results in a response greater than expected (i.e., greater than the sum of their individual effects) [22]. This principle is applied in ameliorating the current antibiotherapy system. When potential bio-compounds are co-dosed with antibiotics, the activity of the antibiotics is enhanced towards the bacteria that were initially developing resistance [23]. We aimed to further investigate the most active microbial inhibitor (BTE) defined by antimicrobial assay for its potentiating activity with three commercial antibiotics: chloramphenicol, ciprofloxacin and streptomycin. To determine the effects of antibiotics used in combination with BTE as synergistic, partial synergy, additive, antagonistic or indifferent, FICI was calculated. The FIC and FICI values are summarized in Table 3.

Most combinations exhibited non-interactive interactions, while no combinations displayed fully synergistic interactions. However, combining BTE with ciprofloxacin and streptomycin resulted in a partial synergistic relationship against MRSA and *P. aeruginosa*, respectively (FICI: 0.62). The combination of BTE: chloramphenicol presented an antagonistic interaction with *E. coli* (FICI: 5). Our findings showed that BTE could partially potentiate ciprofloxacin against MRSA and streptomycin against *P. aeruginosa*. It is understood that we can barely rely on existing antibiotics to control infections due to the resistance they have developed against numerous microbes. Clinicians should therefore prescribe antibiotics carefully and when necessary to avoid deteriorating the current situation. It is reported that the prescription of 30–60% of antibiotics in intensive care units has been found to be unnecessary, inappropriate or suboptimal [24].

### 2.4. BRA and BFA Extracts Inhibit ZIKV Infection at Non-Cytotoxic Concentrations

Cytotoxicity testing is crucial in assessing and validating the safe use of medicinal plants as traditional medicine and also fills in as a guide to search for new biomolecules with promising biological activities [25]. Hence, prior to the assessment of the anti-ZIKV activity of *B. gymnorhiza* extracts, maximal non-cytotoxic concentrations (MNTC) on human lung epithelial cell lines (A549) were determined. We opted to evaluate the cytotoxic effects of the methanolic, ethyl acetate, decoction and aqueous extracts of *B. gymnorhiza* against A549 cells as it is the most common cancer leading to death [26]. Consequently, it is of prime concern for the scientific community to search for an effective treatment strategy to eradicate this disease burden.

**Figure 1:**
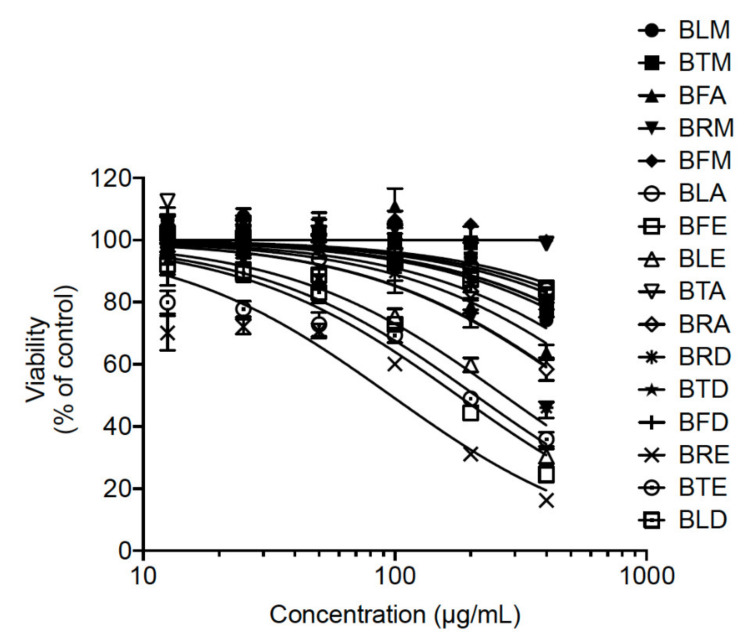
Cytotoxicity of *B. gymnorhiza* extract on A549 cell lines. A549 cells were incubated with different concentrations of *B. gymnorhiza* extract for 48 h. Cell viability was determined using the metabolic activity by MTT assays. Results are given as means ± SEM of four independent experiments and expressed as relative values in contrast to untreated cells.

**Figure 2:**
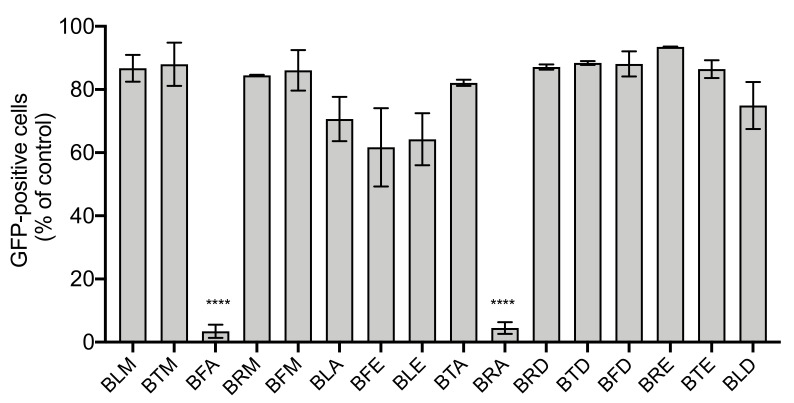
BFA and BRA exert antiviral activity against ZIKV. A549 cells were infected with ZIKV^GFP^ at MOI of 2 in the presence of *B. gymnorhiza* extracts at 200 µg/mL. After 24 h of infection, flow cytometric analysis of GFP fluorescence was conducted. The results are given as means ± SD of three independent experiments and expressed as relative values in contrast to untreated infected cells. **** *p* < 0.0001 indicates significant differences in the extracts.

**Figure 3:**
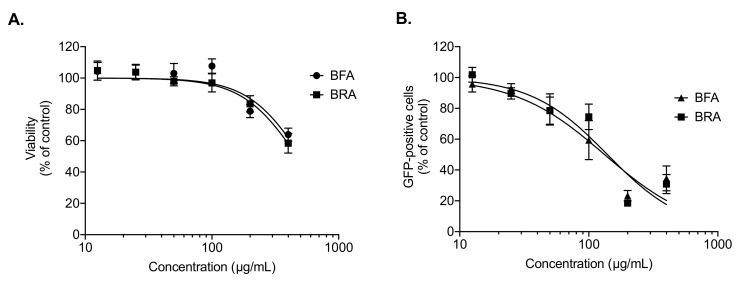
BFA and BRA extracts exert a dose–response antiviral activity against ZIKV. (**A**) A549 cells were incubated with *B. gymnorhiza* extracts (400 to 12.5 µg/mL) for 72 h. Cell viability was assessed by MTT assay. Results are given as means ± SD of four independent experiments and expressed as relative values compared to vehicle. (**B**) A549 cells were infected with ZIKV^GFP^ at MOI of 2 in presence of *B. gymnorhiza* extracts (400 to 12.5 µg/mL). After 24 h of infection, flow cytometric analysis of GFP fluorescence was conducted. The results are given as means ± SD of four independent experiments and expressed as relative value in contrast to untreated infected cells.

The A549 cells were incubated with increasing concentrations of extracts for 48 h using MTT assay. From the MTT results, cytotoxic effects on A549 cells increased as the concentration of extracts increased except in the case of the BRM extract, as shown in Figure 1. From the latter figure, it was observed that the cell viability began to decrease drastically at concentrations greater than 200 µg/mL. Therefore, *B. gymnorhiza* extracts at a concentration of 200 µg/mL were prepared to test anti-ZIKV activity. To be able to monitor viral infection in A549 cells by flow cytometry whereby ZIKV can replicate efficiently, a chimeric molecular clone of the African strain of the Zika virus expressing a GFP reported gene (ZIKV^GFP^) was used. As such, A549 cells were infected with ZIKV^GFP^ in the presence of *B. gymnorhiza* extracts prepared at the MNTC of 200 µg/mL. Figure 2 shows that among the 16 tested extracts, only BRA and BFA significantly (*p* < 0.0001) inhibited ZIKV infection at the non-cytotoxic concentration of 200 µg/mL. Figure 3 clearly illustrates that cell viability decreased significantly and low viral growth was detected at a concentration of 200 µg/mL of both BRA and BFA. The concentrations inhibiting 50% of cell viability (CC50) for BRA and BFA were determined as 520 and 470 µMg/mL, respectively (Table 3). The concentration that inhibits 50% ZIKV infection (IC50) was also calculated as 130 and 140 µg/mL for BRA and BFA, respectively (Table 4). The observed activity can be linked to the phytochemical profiles of these extracts (Please refer to Supplementary Materials). Past evidence suggested that combinations of antiviral drugs originating from several classes having different mechanisms of action that act on the various phases of the life cycle of a virus are anticipated to be synergistic [27]. Hence, herein, we hypothesized that the interactions that exist between the biomolecules present in BRA and BFA can be synergistic. Collectively, these findings revealed that BRA and BFA can effectively inhibit ZIKV infection in A549 cells, demonstrating the ability of these extracts to be considered as a source of natural antiviral phytocompounds.

### 2.5. Viral Inactivation Assay Shows That BRA and BFA Prevent ZIKV Entry in A549 Cells

**Figure 4:**
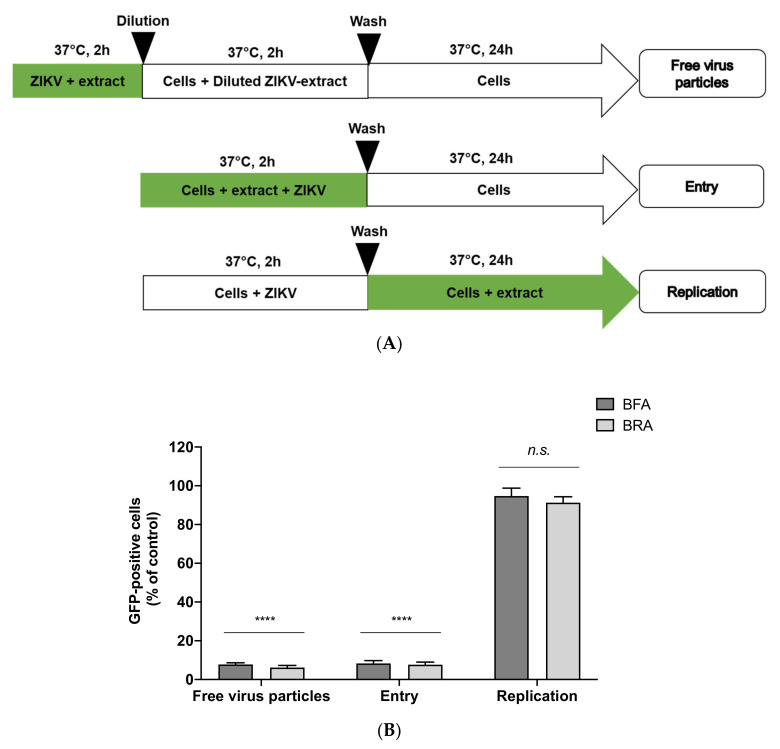
*B. gymnorhiza* extracts inhibit ZIKV entry in human cells. (**A**) Schematic diagram representing the time-of-drug-addition assay. The green segments in the arrows show the presence of extract during infection. (**B**) Flow cytometric analysis of GFP expression in A549 cells infected with ZIKV^GFP^ at MOI of 2 PFU/cell under the experimental conditions as illustrated in (**A**). Results are given as means ± SD of three independent experiments and expressed as relative values in contrast to the control. **** *p* < 0.0001 indicates significant differences in the extracts; *n.s.* = not significant.

Time-of-drug-addition assay was used to determine which stages of ZIKV infection were affected by the BRA and BFA extracts (Figure 4A). To assess the effects on the viral entry phase, A549 cells were treated with extracts (BRA and BFA) at the non-cytotoxic concentration of 100 µg/mL were co-added simultaneously to ZIKV during 2 h kept at 37 °C (Figure 4A: entry). To assess effects in the post-infection phase, A549 cells were initially infected with ZIKV for 2 h and then treated with BRA and BFA. The fluorescence intensity representing viral replication was detected in less than 10% of cells (Figure 4B: entry). Nonetheless, no remarkable activity was noticed when BRA and BFA were added 2 h post adsorption (Figure 4B: replication). These data showed that the anti-ZIKV activity of BRA and BFA was not resolved by the inhibition in the replication phase but instead by the inhibition that occurred in the first steps of the viral life cycle, i.e., the entry phase. To probe whether BRA and BFA extracts counteract viral infection in the A549 host cells, ZIKV^GFP^ particles were mixed with the extracts, allowed to incubate for 2 h at 37 °C and then diluted 50-fold before being added to the cells for infection. With this dilution, the concentrations of the extracts dropped below their therapeutic concentrations and blocked ZIKV from binding to the surface of A549 cells.

Our data demonstrated that treating the two *B. gymnorhiza* extracts with ZIKV^GFP^ resulted in a 90% reduction in GFP-positive cells after an incubation of 24 h in contrast to A549 cells infected with untreated ZIKV^GFP^ (Figure 4A, B: free virus particles). Therefore, from these data, we suggest that the biomolecules present in BRA and BFA extracts might have interacted with the ZIKV-free particles, which consequently prevented the host cells from being infected. We presume that the inhibition at entry level could probably be due to a direct interaction between polyphenols present in the extracts and the lipid membrane (outer membrane) of the flavivirus. In fact, this type of interaction was also reported by epigallocatechin gallate and delphinidin [28,29]. The types of phytochemicals present in BRA and BFA originating from several classes such as tannins (procyanidin B and C), flavonoids (catechin, epicatechin, epigallocatechin) and phenolic acids (caffeic acid, quinic acid, ferulic acid) are regarded as promising antiviral leads [30,31]. Taken together, we suggest that the leading anti-ZIKV activities of BRA and BFA are assisted by the interaction between phytochemicals in the extracts and ZIKV-free particles.

### 2.6. In Silico Docking Analysis

The entry of ZIKV into a host cell is mostly governed by the E glycoprotein of the virus and therefore represents a premier target for developing drugs and vaccines against ZIKV [32]. Following in vitro analysis, we chose to dock the compounds bruguierol A, brugierol, quinic acid, citric acid, naringenin, neochlorogenic acid, cryptochlorogenic acid, phloretin and procyanidin B and C against the E protein of ZIKV since several lines of investigations have already documented their viral inhibitory activity in vitro [33,34,35,36,37].

**Figure 5:**
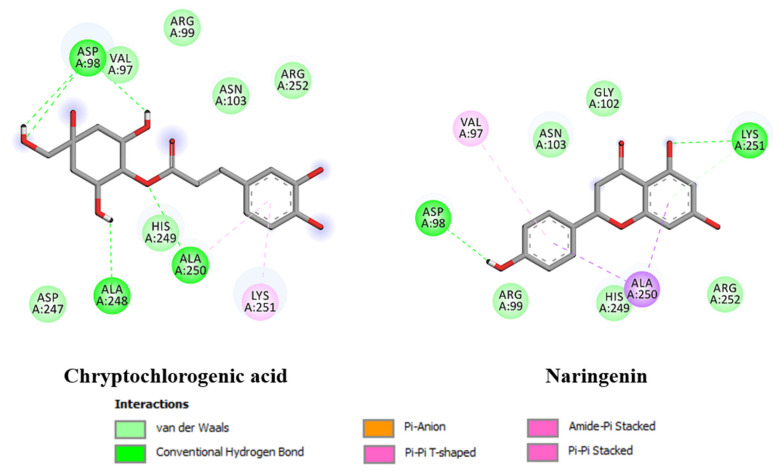
Non-bonding interactions of highest binding affinity against ZIKV envelope protein using docking calculations.

The docking simulation was therefore achieved using the envelope protein and the docking results are listed in Table 5. The range of the binding free energy, which represents the binding affinity of the compound, was between −3.11 kcal/mol for brugierol and −5.44 kcal/mol for cryptochlorogenic acid in the case of the envelope protein, which shows a potential inhibition activity or the natural source of these compounds against ZIKV. Indeed, cryptochlorogenic acid showed the highest binding affinity among the ten studied compounds against the envelope protein followed by naringenin. The calculated inhibition constant, Ki, of these compounds followed the same order. Obviously, the numerical value of the calculated binding affinity is attributed to the nonbonding interactions of the ligand and the protein active site. Among the different types of nonbonding interactions, hydrogen bond was the strongest interaction followed by π–π stacking. Cryptochlorogenic acid formed numerous hydrogen bonds with different amino acids at the active site and π–π interaction. Figure 5 illustrates the nonbonding interactions of the top compounds with the active site of the envelope protein of ZIKV.

**Figure 6:**
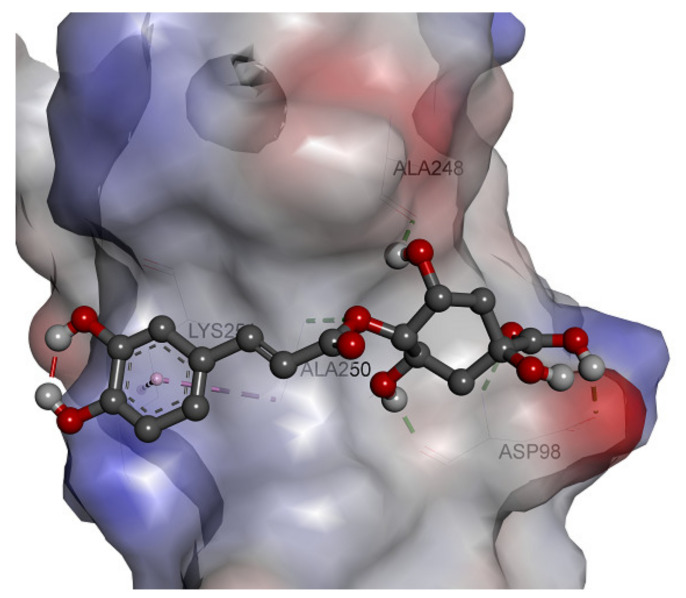
Three-dimensional structure of cryptochlorogenic acid at the active site of ZIKV envelope protein using docking calculation.

The 3D structure of the complex formed between cryptochlorogenic acid and the active site of the target is shown in Figure 6. The surface of the active site is represented by the charge distribution of the amino acids at the active site. The red color represents the negative charge, while the blue color of the amino acids shows negative charge. As shown in Figure 6, cryptochlorogenic acid is trapped between two negatively charged amino acids, Ala248 and Asp98, where most of the hydrogen bonds are formed in the case of the envelope protein.

### 2.7. In Silico ADME Analysis of Cryptochlorogenic Acid

**Figure 7:**
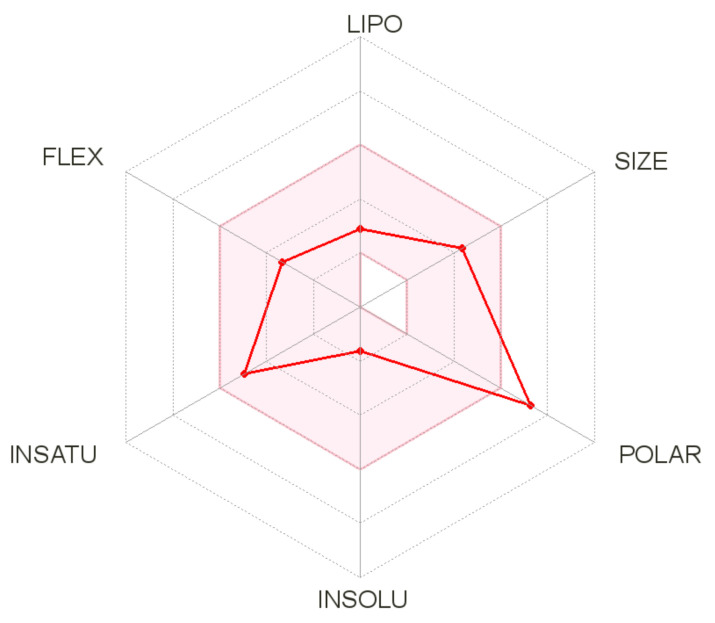
The bioavailability radar plot of cryptochlorogenic acid. The pink area represents the optimal range for each property (lipophilicity: XLOGP3 between −0.7 and +5.0, size: MW between 150 and 500 g/mol, polarity: TPSA between 20 and 130 Å2, solubility: log S not higher than 6, saturation: fraction of carbons in the sp3 hybridization not less than 0.25, and flexibility: no more than 9 rotatable bonds). Cryptochlorogenic acid is predicted not to be orally bioavailable because it is too polar.

The promising results of docking analysis allowed us to explore the ADME characteristics of cryptochlorogenic acid, which showed the highest binding free energy among the 10 compounds docked. ADME analysis involves the prediction of different properties of compounds, namely oral absorption, bioavailability, the ability to penetrate the blood–brain barrier (BBB), distribution and excretion. The aforementioned properties offer important information on the dose, route of administration and safety of the compound of interest. ADME analysis involves descriptors, namely (i) BBB penetration, (ii) gastro intestinal (GI) absorption, (iii) water solubility, (iv) cytochrome P450 (CYP) and p-glycoprotein (p-gp). Predicted descriptors are given in Table 6. The results showed that cryptochlorogenic acid is not a good BBB permeant; thus, this compound may not be safe for the central nervous system [38]. For a molecule to be considered non-drug-like, the molecule should fall entirely inside the pink area of the bioavailability radar plot [39]. The radar plot illustrated in Figure 7 indicates that cryptochlorogenic acid is not orally bioavailable because it is too polar. According to both topological methods such as ESOL [40] and Ali et al. [41], cryptochlorogenic acid is significantly soluble, which could ease its formulation. CYP are isoenzymes essential in drug elimination via metabolic biotransformation. The inhibition of these isoenzymes reduces the metabolism of a drug, leading to toxic or unwanted side effects due to the accumulation of the drug or its metabolites [39]. Our ADME data showed that cryptochlorogenic acid did not inhibit any CYP isoenzymes, thus the excretion of the molecule is easy.

## 3. Materials and Methods

### 3.1. Plant Materials

The different parts of *B. gymnorhiza* (leaves, twigs, roots and fruits) were collected along the coastline of Bambous Virieux in Grand Port, Mauritius (GPS: 20°20′13.88″ S; 57°45′54.99″ E) during the rainy summer season on the 16 April 2018. The plant identification was verified by the herbarium of Mauritius Sugarcane Industry and Research Institute, Réduit, Mauritius, where a voucher specimen (MAU 0029125) was deposited.

### 3.2. Preparation of Extracts

The collected plant materials were carefully washed under running tap water to remove surface detritus and sands and were allowed to dry in the shade. When a constant mass was recorded, each dried plant part (leaves, roots, twigs, fruits) was powdered. The samples were prepared via two different types of extraction methods, namely maceration and decoction. Each plant part (50 g) was exhaustively macerated in 500 mL of three different solvents, namely distilled water, 70% methanol and ethyl acetate, while decoctions were prepared by allowing 50 g of plant parts to boil in 200 mL distilled water for 30 min. The extracts were filtered and concentrated in a rotary evaporator at 37 °C. The concentrated extracts were then lyophilized and preserved at +4 °C until further investigation.

### 3.3. Antimicrobial Assay

The microorganisms *Escherichia coli* ATCC 25922, *Pseudomonas aeruginosa* ATCC 27853, *Klebsiella pneumoniae* ATCC 70603, methicillin-resistant *Staphylococcus aureus* ATCC 43300 (MRSA), *Salmonella enteritidis* ATCC 13076, *Sarcina lutea* ATCC 9341, *Proteus mirabilis* ATCC 25933, *Bacillus cereus* ATCC 11778 and *Candida albicans* ATCC 26555 were used to evaluate the antimicrobial properties of *B. gymnorhiza* extracts. The preparation of bacterial cultures, adjustment of McFarland density and bacterial inoculum for assays were conducted as detailed in Koc and Uysal [42]. The broth micro dilution method was carried out according to our previous published papers [43,44] with minor modifications.

### 3.4. Antibiotic Potentiating Assay

The extract (BTE) with the lowest MIC value was selected to conduct antibiotic potentiating activity. A known volume of the stock solution of BTE (25 mg/mL) was combined with commercial antibiotics, namely ciprofloxacin (CIP) (0.1 mg/mL), chloramphenicol (CHL) (1 mg/mL) and streptomycin (STR) (1 mg/mL), in a ratio of 1:1. The assay was carried out using broth micro dilution method as described in Section 2.4. The different antibiotics (CIP, CHL and STR) were used alone as positive controls and MHB as negative control. The MIC values were recorded and the results of the combined effects of the antibiotics and BTE were calculated and expressed in terms of the fractional inhibitory concentration index (FICI), which was denoted by the following Equations (1)–(3):

FIC _extract_ = MIC of extract in combination/MIC of extract alone (1)

FIC _antibiotic_ = MIC of antibiotic in combination/MIC of antibiotic alone (2)

FICI = FIC _extract_ + FIC _antibiotic_ (3)

FIC _extract_ is the fractional inhibitory concentration of the extract and FIC _antibiotic_ is the fractional inhibitory concentration of the antibiotic used.

The FICI values were interpreted as follows: synergistic (≤0.5); partial synergy (0.5–0.75); additive (0.76–1.0); indifferent (non-interactive) (>1.0–4.0); antagonistic (>4.0) [45,46].

### 3.5. Cell Cultures

Human lung epithelial A549 cells (ATCC, CCL-185, Manassas, VA, USA) were cultured in Eagle minimum essential medium (MEM) supplemented with 5% heat-inactivated fetal bovine serum (FBS), 2 mmol/L L-glutamine, 1 mmol/L sodium pyruvate, 100 U/mL of penicillin, 0.1 mg/mL of streptomycin and 0.5 µg/mL of fungizone (PAN Biotech, Aidenbach, Germany) under a 5% carbon dioxide atmosphere kept at a temperature of 37 °C. The clinical isolate PF-25013-18 of ZIKV has been previously described [47]. A GFP-expressing ZIKV (ZIKV^GFP^) was derived from a molecular clone ZIKV^MC-MR766NIID^ (ZIKV^GFP^), which is the ancestral Zika virus strain MR766-NIID [48]. A549 cells were infected with a multiplicity of infection (MOI) of 2. ZIKV strains were amplified on Vero cells. Virus stocks were cultured and titrated on Vero cells using plaque-forming assay.

### 3.6. MTT Assay

The prepared extracts of *B. gymnorhiza* were tested for their cytotoxic effect against the human lung epithelial cell lines (A549) using 3-[4,5-dimethylthiazol-2-yl]-2,5-diphenyltetrazolium bromide (MTT) assay. Cells were cultured in 96-well plates at a density of 1.0 × 10^4^ cells per well. The cells were treated with plant samples of different concentrations ranging from 400 to 12.5 µg/mL and then allowed to incubate for 48 h at 37 °C. Post incubation period, the cells were washed with PBS 1× and then 120 µL of culture medium mixed with 5 mg/mL MTT solution was used to wash the cell monolayer. After a period of incubation time of 2 h was applied, MTT was removed and 50 µL dimethyl sulfoxide (DMSO) was added to solubilize the formazan crystals [49].

### 3.7. Virus Inactivation Assay

The GFP-expressing ZIKV (ZIKV^GFP^) (2 × 10^5^ PFU) was mixed with plant samples (200 µg/mL) and allowed to incubate for 2 h at 37 °C to investigate the direct influence of the samples on viral infectivity. A dilution of 50-fold (final virus concentration, 1 PFU/well) was conducted on the mixture with MEM (10% FBS) to attain the subtherapeutic concentrations of the respective samples. The resulting mixture was then pipetted in 6-well plates containing the monolayer of A549 cells. A control was prepared by mixing ZIKV^GFP^ with extract, diluted 50-fold without incubation period and added to the cells for infection [50]. After an adsorption that lasted for 2 h and was maintained at 37 °C, the diluted inocula were removed, and the cells were washed 2× with PBS. The plates were further incubated at 37 °C for 24 h with fresh medium prior to a flow cytometric analysis.

### 3.8. Flow Cytometry Assay

Cells were fixed with 3.7% paraformaldehyde (PFA) in PBS for 20 min, and washed 2× with PBS prior to flow cytometric analysis by Cytoflex (Beckman, CA, USA). Results were analyzed using cytexpert software [51].

### 3.9. In Silico Docking Analysis

Ten compounds were further studied theoretically using docking simulation. Our target was to find the binding affinity, inhibition constant and the intramolecular interactions of the detected compounds against ZIKV envelope protein and protease enzyme. The crystal structure of the envelope protein and protease enzyme is available online in RCSB PDB (www.rcsb.org, accessed on: 11 January 2021) with the pdb code 5H4I for the protease enzyme and 5JHM for the envelope protein. Three-dimensional structures of the potential inhibitors were downloaded from PubChem (www.pubchem.ncbi.nlm.nih.gov, accessed on: 11 January 2021) and ChemSpider (http://www.chemspider.com/, accessed on: 11 January 2021) databases. The structures were initially optimized using AM1 semiempirical method [52] and the atomic charges were fixed and saved in mol2 format. In order to prepare the protein crystal structure for docking calculations, the co-crystalized molecules such as water molecules were removed. Autodock 4 (Molinspiration database: http://www.molinspiration.com, accessed on: 11 January 2021) was selected for the docking simulation. Kollman united atom charges were added to neutralize the protein and a grid box of 60 × 60 × 60 dimensions with 0.375 Å distance was used. Lamarckian genetic algorithm was used to evaluate the 250 conformations of the inhibitors at the protein active site. Discovery studio 5.0 visualizer was used to analyze the intramolecular interactions and prepare the figures.

### 3.10. In Silico ADME Analysis of Cryptochlorogenic Acid

The ADME of cryptochlorogenic acid was predicted using swissADME online software (http://www.swissadme.ch/index.php, accessed on: 15 February 2021) [39].

### 3.11. Statistical Analysis

For comparison among samples, one-way ANOVA test was performed. All values were given as mean ± SD of at least three independent tests. All statistical analyses were conducted using GraphPad prism software (version 8.0; GraphPad software, La Jolla, CA, USA). Levels of significance are indicated on the figures as follows: * *p* < 0.05; ** *p* < 0.01; *** *p* < 0.001, **** *p* < 0.0001, *n.s* = not significant.

## 4. Conclusions

No effective therapy against ZIKV infections nor new antibiotics to combat antimicrobial resistance are presently available. Although BRA and BFA were among the least potent antimicrobial extracts, they were found to be the most potent, promising and natural anti-ZIKV agents with low cytotoxicity at a concentration of 200 µg/mL against A549 cells. Further data collected in this work underscore the mangrove plant *B. gymnorhiza* from Mauritius as a promising source for the development of natural and safe antiviral agents to confront ZIKV infections. As far as our literature search could reach, our report is the first in scrutinizing a mangrove plant from Mauritius for its anti-ZIKV activity, and it is thus added to the list of medicinal plants of the Mascarene Islands having the ability to inhibit ZIKV at the entry phase of the life cycle of the virus. From the results of docking analysis, we presumed that among the 10 compounds selected, cryptochlorogenic acid has contributed the most in inhibiting the entry of ZIKV into the host cell. In terms of antibiotic potentiating activity, BTE could partially potentiate ciprofloxacin against MRSA and streptomycin against *Pseudomonas aeruginosa*. However, it is important to bear in mind that the extract to antibiotic ratio used in our present study was 1:1. Working at different ratios such as 3:7 or 7:3 using the variable ratio analysis method could show interesting results in the future. Cryptochlorogenic acid is not a safe drug to administer, though it is easy to synthesize. Thus, discovery to market attrition is reduced. It may be instead administered as a slow intravenous infusion. In future research, it would be of great scientific concern to identify which compounds were responsible for the anti-ZIKV activity of *B. gymnorhiza* and also to confirm our hypothesis on whether the observed activity was a result of a synergistic interaction.

## Figures and Tables

**Table 1:** Yield and code with respect to extract prepared.

Method	Plant Part	Code	Yield (%)
Decoction	Leaf	BLD	18.70
	Root	BRD	7.38
	Twig	BTD	7.78
	Fruit	BFD	8.24
Maceration: Water	Leaf	BLA	11.04
	Root	BRA	4.28
	Twig	BTA	4.26
	Fruit	BFA	11.12
Maceration: Ethyl Acetate	Leaf	BLE	1.88
	Root	BRE	0.46
	Twig	BTE	1.06
	Fruit	BFE	0.66
Maceration: Methanol	Leaf	BLM	12.62
	Root	BRM	9.60
	Twig	BTM	8.26
	Fruit	BFM	14.28

**Table 2:** Antimicrobial activity of *B. gymnorhiza* extracts (MIC50 values in mg/mL) against nine microorganisms.

Strains	MIC50 Values of *B. gymnorhiza* Extracts (mg/mL)																
	BLM	BRM	BTM	BFM	BLE	BRE	BTE	BFE	BLA	BRA	BTA	BFA	BLD	BRD	BTD	BFD	Gentamicin (µg/mL)
*Escherichia coli* ATCC 25922	-	1.56	-	-	-	0.78	0.19	0.78	-	-	-	6.25	-	-	-	3.12	0.312
*Pseudomonas aeruginosa* ATCC 27853	0.78	-	0.78	0.78	0.78	0.78	0.19	0.39	-	-	-	3.12	-	1.56	3.25	0.78	0.039
*Klebsiella pneumoniae* ATCC 70603	-	1.56	-	1.56	-	1.56	0.39	0.78	-	-	-	-	-	-	-	1.56	1.25
*Staphylococcus aureus* ATCC 43300 (MRSA)	3.12	0.39	0.78	0.39	3.12	0.78	0.19	0.78	-	0.39	6.25	0.39	6.25	0.39	0.78	1.56	0.078
*Salmonella enteritidis* ATCC 13076	-	1.56	-	1.56	-	1.56	0.39	0.78	-	-	-	3.12	-	3.12	-	1.56	0.078
*Sarcina lutea* ATCC 9341	1.56	-	-	1.56	-	1.56	0.39	0.78	-	-	-	3.12	-	3.12	-	1.56	0.039
*Proteus mirabilis* ATCC 25933	-	0.78	1.56	0.39	-	0.78	0.39	1.56	-	3.12	-	1.56	-	0.78	3.12	1.56	0.312
*Bacillus cereus* ATCC 11778	-	-	-	-	-	1.56	0.39	0.78	-	-	-	-	-	-	-	3.12	<0.039
*Candida albicans* ATCC 26555	-	-	-	1.56	-	1.56	0.39	0.78	-	-	-	-	-	6.25	-	3.12	0.312

**Table 3:** Antibiotic potentiating activity of *Bruguiera* twig ethyl acetate extract (BTE).

BTE/Antibiotic Combination (1:1)	*E. coli*		*P. aeruginosa*		MRSA	
	FIC	FICI	FIC	FICI	FIC	FICI
BTE	0.13	1.13 ^b^	0.25	2.25 ^b^	0.13	0.62 ^a^
CIP	1		2		0.49	
BTE	1	5 ^c^	1	1.25 ^b^	0.26	2.26 ^b^
CHL	4		0.25		2	
BTE	0.26	2.26 ^b^	0.13	0.62 ^a^	0.26	1.26 ^b^
STR	2		0.49		1	

FIC: fractional inhibitory concentration; FICI: fractional inhibitory concentration index; CHL: chloramphenicol; CIP: ciprofloxacin; STR: streptomycin; *E. coli*: *Escherichia coli*; *P. aeruginosa*: *Pseudomonas aeruginosa*; MRSA: methicillin-resistant *Staphylococcus aureus.* ^a^ Partial synergy; ^b^ Indifferent; ^c^ Antagonistic.

**Table 4:** Cytotoxicity and anti-ZIKV activity of *B. gymnorhiza* extracts.

Extract	CC_50_ (µMg/mL) ^a^	IC_50_ (µg/mL) ^b^	SI ^c^
BFA	520	130	4.0
BRA	470	140	3.3

Cytotoxic concentration (CC) and inhibitory concentration (IC) were calculated by performing nonlinear regression followed by constructing sigmoidal dose–response curves from Figure 3A,B. ^a^ concentration with 50% cell viability; ^b^ concentrations that inhibited infection by 50%; ^c^ Selectivity Index (CC_50_/IC_50_).

**Table 5:** Binding free energy (kcal/mol), calculated inhibition constant (K_i_) and protein–ligand interactions of the listed compounds against ZIKV envelope protein using docking calculations.

Compound	Binding Free Energy	Inhibition Constant	Protein-Ligand Interactions
Brugierol	−3.11	5.3 nM	Lys251 (HB), Asn103, Ala250, His249, Arg252
Bruguierol A	−4.31	689.9 µM	Gly102 (HB), Arg252, His249, Lys251, Ala250 (HB) and (π- π)
Cryptochlorogenic Acid	−5.44	102.1 µM	Asp98 (HB), Ala248 (HB), Ala250 (HB), Lys251 (π- π), Val97, Asn103, His249.
Citric acid	−3.31	3.75 mM	His249 (HB), Ala248 (HB), Asn103 (HB), Asp98 (HB), Val97, Ala250.
Naringenin	−5.07	192.7 µM	Lys251 (HB), Val97, Asp98 (HB), Ala250, Asn103, His249.
Neochlorogenic Acid	−4.07	1.1 mM	Asp98 (HB), Val97, Ala250, Asn103, Arg99, Lys110, Gly109, Phe108.
Phloretin	−4.65	393.5 µM	Ala250 (HB), Lys251 (HB), Ala248 (HB), Val97, His249, Asn103, Gly102.
Procyanidin B	−4.57	447.6 µM	Ala248 (HB), Ala250 (HB), Arg99 (HB), Gly102 (HB), Asn103 (HB), Arg252.
Procyanidin C	−4.09	1.0 mM	Ala248 (HB), His249 (HB), Asp98 (HB), Lys110, Val97, Ala250, Asn103, Gly102.
Quinic acid	−4.35	644.0 µM	Asp98 (HB), Ala248 (HB), His249 (HB), Val97, Lys110, Asn103.

Noted: gray—the two compounds exhibited the highest binding affinity.

**Table 6:** Predicted ADME for cryptochlorogenic acid.

Physicochemical Properties		Lipophilicity		Water Solubility	
Formula	C16H18O9	Log P_o/w_ (iLOGP)	1.23	Log S (ESOL) = −1.62	Very soluble
MW	354.31 g/mol	Log P_o/w_ (XLOGP3)	−0.42	Log S (Ali) = −2.58	Soluble
No. of heavy atoms	25	Log P_o/w_ (WLOGP)	−0.75	Log S (SILICOS-IT) = 0.40	Soluble
No. aromatic heavy atoms	6	Log P_o/w_ (MLOGP)	−1.05		
Fraction Csp^3^	0.38	Log P_o/w_ (SILICOS-IT)	−0.6		
No. rotatable bonds	5	Mean Log P_o/w_	−0.32		
No. H-bond acceptors	9				
No. H-bond donors	6				
Molar refractivity	83.5				
TPSA	164.75 Å2				
**Pharmacokinetics**		**Drug Likeness**		**Medicinal Chemistry**	
GI absorption	Low	Lipinski	Yes; 1 violation: NH or OH > 5	PAINS	1 alert: catechol_A
BBB permeant	No	Ghose	No; 1 violation: WLOGP < −0.4	Lead-likeness	No; 1 violation: MW > 350
P-gp substrate	No	Veber	No; 1 violation: TPSA > 140	Synthetic accessibility	4.13
CYP1A2 inhibitor	No	Egan	No; 1 violation: TPSA > 131.6		
CYP2C19 inhibitor	No	Bioavailability Score	0.11		
CYP2C9 inhibitor	No				
CYP2D6 inhibitor	No				
CYP3A4 inhibitor	No				
Log K_p_ (skin permeation)	−8.76 cm/s				

MW: Molecular weight; TPSA: Topological polar surface area; Log P_o/w_: Partition coefficient between n-octanol and water; GI: Gastrointestinal; BBB: Blood–brain barrier; CYP: Cytochrome P450; PAINS: Pan assay interference compounds; Synthetic accessibility score: 1 (very easy) to 10 (very difficult.).

## Data Availability

Not applicable.

## Supplementary Materials

The following are available online. Dataset containing all the phytochemicals putatively annotated by means of ultra-high-performance liquid chromatography/electrospray ionization tandem mass spectrometry (UHPLC- ESI-MS/MS).

## Abbreviations

BFA	*Bruguiera* fruit aqueous
BLA	*Bruguiera* leaf aqueous
BRA	*Bruguiera* root aqueous
BTA	*Bruguiera* twig aqueous
BFD	*Bruguiera* fruit decoction
BLD	*Bruguiera* leaf decoction
BRD	*Bruguiera* root decoction
BTD	*Bruguiera* twig decoction
BFE	*Bruguiera* fruit ethyl acetate
BLE	*Bruguiera* leaf ethyl acetate
BRE	*Bruguiera* root ethyl acetate
BTE	*Bruguiera* twig ethyl acetate
BFM	*Bruguiera* fruit methanolic
BLM	*Bruguiera* leaf methanolic
BRM	*Bruguiera* root methanolic
BTM	*Bruguiera* twig methanolic
